# Constitutional Relations of the Teeth

**Published:** 1876-01

**Authors:** D. C. Hawxhurst


### CONSTITUTIONAL RELATIONS OF THE TEETH.

BY D. C. HAWXHURST.

*Read upon retiring from the Presidency of the Michigan State Dental Association, October, 1874.*

*Gentlemen:*—I have hastily thrown together some considerations by which I would indicate to you my belief that dental surgery is a distinct and proper branch of medicine, and that the sympathetic diseases that certainly run abreast in the teeth and in the body demand of us an acquaintance with the great principles of medicine.

I am aware that many of you already hold this view substantially, and that one of your ex-presidents has read before this body a carefully prepared paper treating of dental surgery as a special branch of medicine. But as a profession we are not yet up to this standard, and the subject will bear further discussion.

I have, therefore, determined to call your attention to the morbid connections existing between the teeth and the rest of the body, and ask you to consider with me how far we as practitioners of a specialty are bound to acquaint ourselves with the entire range of vital phenomena, both in health and disease.

And the first point that I would ask you to consider is the essential unity of the human body, the essential unity of the vital force that pervades it, the identity of diseased action in large classes of disorders, and the identity of those vital changes which look toward health.

The animal economy, it is true, when considered as a mechanism, is seen to be made up of an assemblage of apparatuses, each looking toward a distinct end; as the circulatory, the digestive, the pulmonary, the excretory systems, the reproductive and the nervous. But these different mechanisms are inextricably combined, "knit by the closest bonds of sympathy," into a single organic whole which feels in all its parts every serious local injury, and which, through the nervo-vital and sanguineous fluids, impresses its own conditions, whether of health or disease, on each of its parts.

And so close are the sympathies that play between the organs and functions of the animal economy, that to excite action in one, is often to arouse another; to affect one unfavorably is to start morbid action in another. Thus menstruation being suppressed, it may be performed vicariously by the stomach, the liver being torpid, a diarrhœa may carry off hepatic impurities, or the skin being arrested in its actions, the lungs pour out a fetid breath.

On account of this close sympathy, a current of air on the shoulder will cause the nose to run; a chill applied to the feet will send a neuralgic pain through the temples; irritation of the alimentary tract will cause itching of the nose; croton oil rubbed externally on the abdomen witl purge the intestine; tickling in the Schneiderian membrane will arouse the respiratory muscles to action or precipitate epileptic convulsions.

Some sympathies are normal, as when flavors in the mouth excite the flow of gastric juice in the stomach; some of them are morbid, as when a disordered state of the stomach produces pain in the heart. What the laws of these sympathetic actions are, we can not tell; they are sometimes direct as in the above cases and often indirect, as when action in one organ quells irritation in another.

I have dwelt somewhat on this question of sympathy, be cause I desired to refer to the many morbid symptoms in the body which may be traced to the teeth, or which, existing previously in the body, may gain expression through the teeth or parts connected with them.

Every mother knows how numerous are the sympathetic diseases of first dentition. Disorders of the nervous system, even convulsions, arise from dental irritation; cutaneous eruptions, diarrhœa, costiveness, cough, spasms, irregularity of kidneys, fever and many other difficulties occur.

Dr. Hunter described a case in his work, in which certain urinary symptoms were present every time a new tooth was cut. "This," says he, "happened so regularly, often and constantly that there was little reason to doubt that it was owing to that cause."

The destruction of health that results from diseases of the teeth in adults is equally striking.

I do not expect to call your attention to new facts in this connection; you are all familiar, as practitioners, with those frightful mouths that come to us in anæmic and broken down women. You are only too well acquainted with the ragged aspect of the dental arch, the fetor of breath, the oozing of sanies from the gums, the dental abscesses, the persistent inflammations which attend the teeth, and which often spread through their sockets, and occasionally involve the maxillary bones and their cavities. You have seen in your daily experiences how depraved action has been propagated from diseased structures to healthy ones; how morbid states of the teeth and periostea have spread; how chronic disease, creeping onward among the teeth and sockets, has at last involved the dental arch, the maxillary bones and almost the entire oral cavity in a common ruin.

I ask you to give attention to these extensive lesions on the general health, and to briefly consider, with me, the strong necessity which is upon us of fitting ourselves fully and completely to deal with the diseases of the oral cavity in their sympathetic connections.

The drain upon vitality which a bad mouth may occasion, is very great, and the amount of disease excited in the general system, through the laws of sympathy, is enormous.

First, we have indigestion, caused by disturbed innervation and imperfect mastication. These causes often produce very severe gastric derangements.

What bad results must follow the swallowing of fouled saliva, mucus, sanious discharges from the gums and the drippings from abscesses I have to conjecture. And how much poison may be introduced into the blood of the patient who must constantly live in and breathe the fetor from these excretions, I will not attempt to estimate. How can I more than allude to the subject of neuralgia, arising from lesions in the dental nerves.

I merely mention these things to refresh your recollections of practice and as tending to show what a broad open gateway for the admission of disease into the general system is to be found in the morbid states of the teeth and parts connected.

The general practitioner will not, as a rule, attend to these matters. There is no body to do it but the dentist. And perhaps it is too much to ask of the general surgeon, that he shall be as keen of eye and sure of judgment in these matters as the dentist Besides the surgeon or physician will seldom think of tracing affections of the eye, pectoral symptoms, neuralgia, hysterics or hypochondria, vertigo, suppression of the menses, headache, earache and other affections that are known results of dental disease to regions of the teeth.

So much for the influence of diseased teeth on the general system. And now I desire to offer a word as to the re-action of constitutional vices and disorders upon the teeth.

(To be continued.)

